# Clinicoradiologic diagnosis of a rare type of congenital adrenal hyperplasia: A case report from Nepal

**DOI:** 10.1002/ccr3.5948

**Published:** 2022-06-09

**Authors:** Hensan Khadka, Siddhartha Bhandari, Prakash Dhakal, Suraj Sharma

**Affiliations:** ^1^ Department of Radiology National Academy of Medical Sciences Bir Hospital Kathmandu Nepal; ^2^ Institute of Medicine Tribhuvan University Kathmandu Nepal

**Keywords:** 17‐alpha‐hydroxylase, CAH, congenital adrenal hyperplasia, radiodiagnosis, radiology

## Abstract

Congenital adrenal hyperplasia includes defects in the synthesis of steroid hormones in the adrenal cortex. The implications of this disorder manifest in other genitourinary organs, including ovaries and uterus. The diagnosis may be suspected based on the clinical and radiologic features.

## Graphical abstract

The diagnosis of congenital adrenal hyperplasia may be made on the basis of clinicoradiologic features if enzyme assays or genetic testing are not available.

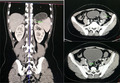

## 1 INTRODUCTION

Congenital adrenal hyperplasia (CAH) is a group of diverse biochemical disorders affecting steroid hormone synthesis in the adrenal cortex. It is characterized by a group of autosomal recessive deficiencies in the enzymes involved in the steroid hormone biosynthesis pathway.^5^

The most common type of CAH is the deficiency of the 21‐hydroxylase enzyme, whereas other rare types include the deficiency of other enzymes, like 17‐alpha‐hydroxylase and 11‐beta‐hydroxylase.^5^ These deficiencies result in decreased formation of products and an accumulation of substrates, thereby contributing to the variety of clinical manifestations seen with the disorder.

Though the most common diagnostic modality used for CAH is the demonstration of low levels of enzymes or products and high levels of precursors, radiology may be of assistance in diagnosing CAH and ruling out adrenal adenoma, especially when combined with clinical features.^9^ We present a case of congenital adrenal hyperplasia with a history of hypertension and occasional per vaginal spotting. Although enzyme assay was not done due to resource constraints, the clinical and radiologic features are suggestive of the 17‐alpha‐hydroxylase deficiency type of CAH. We could not identify any radiologic reporting of such cases in a literature review.

## 2 CASE DESCRIPTION

A 30‐year‐female with a history of hypertension under medications presented with a history of insidious onset progressive generalized body weakness for 3 months. Her menstrual history was significant for irregular scant per vaginal spotting alternating with amenorrhea, both for the last 3–4 years. Her physical examination was insignificant except for mild hypertension of 140/100 mm Hg. She had normal secondary sexual characteristics and external genitalia with no features of virilization. These clinical features were noted, and blood investigations and imaging studies were ordered.

Her blood workup showed the following reports Table 1:

**TABLE 1:** Laboratory reports

Test	Results
Early morning aldosterone level	7.8 ng/dl
Plasma renin activity	0.28 ng/ml/hr
Aldosterone/Renin ratio	27.86 ng/dl per ng/ml/hr
HbA1c	5.8%
Early morning serum cortisol level	4.68 mcg/dl
FSH	22.3 µIU/ml
LH	27.4 mIU/ml

As shown in the table, she had an early morning aldosterone level of 7.8 ng/dl and plasma renin activity of 0.28 ng/ml/hr, both of which were normal as per reference levels in the lying down position. Her resultant plasma aldosterone/renin ratio was 27.86 ng/dl per ng/ml/hr, which was elevated. Her early morning serum cortisol level was slightly low at 4.68 mcg/dl. Her serum follicle‐stimulating hormone (FSH) and luteinizing hormone (LH) concentrations were both elevated and in the post‐menopausal range (FSH 22.3 µIU/ml and LH 27.4 mIU/ml).

**FIGURE 1:**
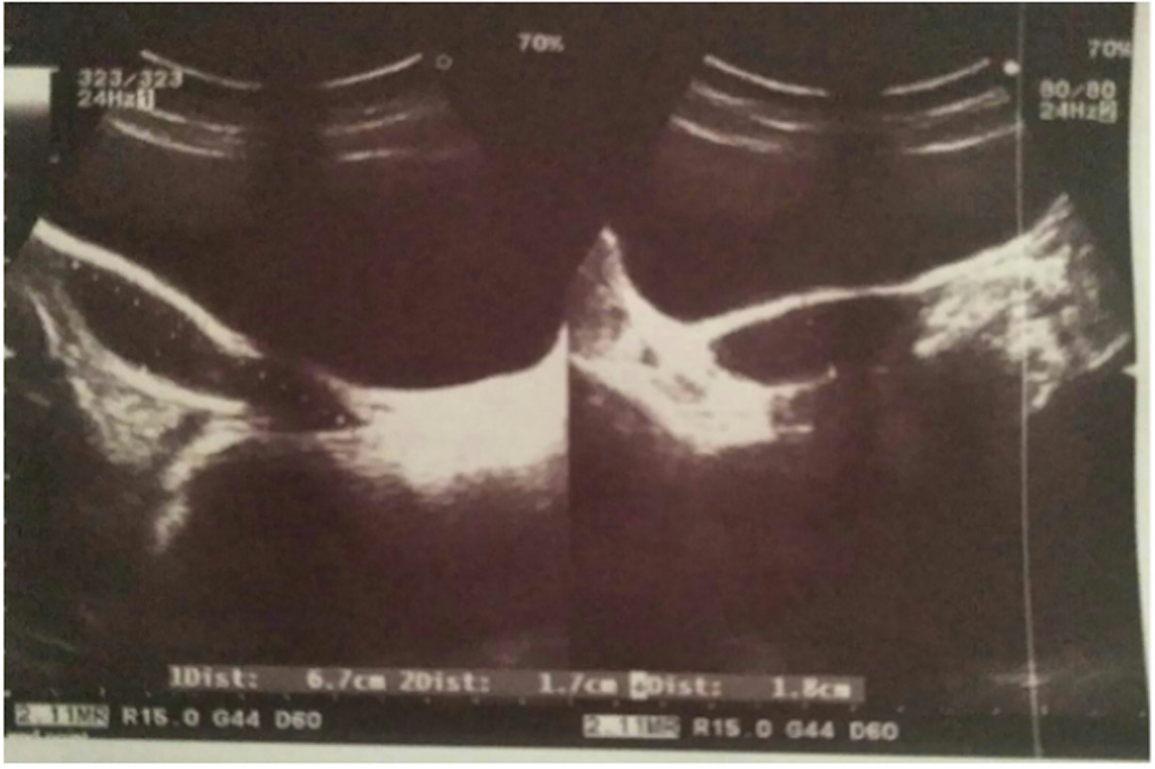
Sagittal and axial transabdominal ultrasound shows a small uterus with thinning of the myometrium

**FIGURE 2:**
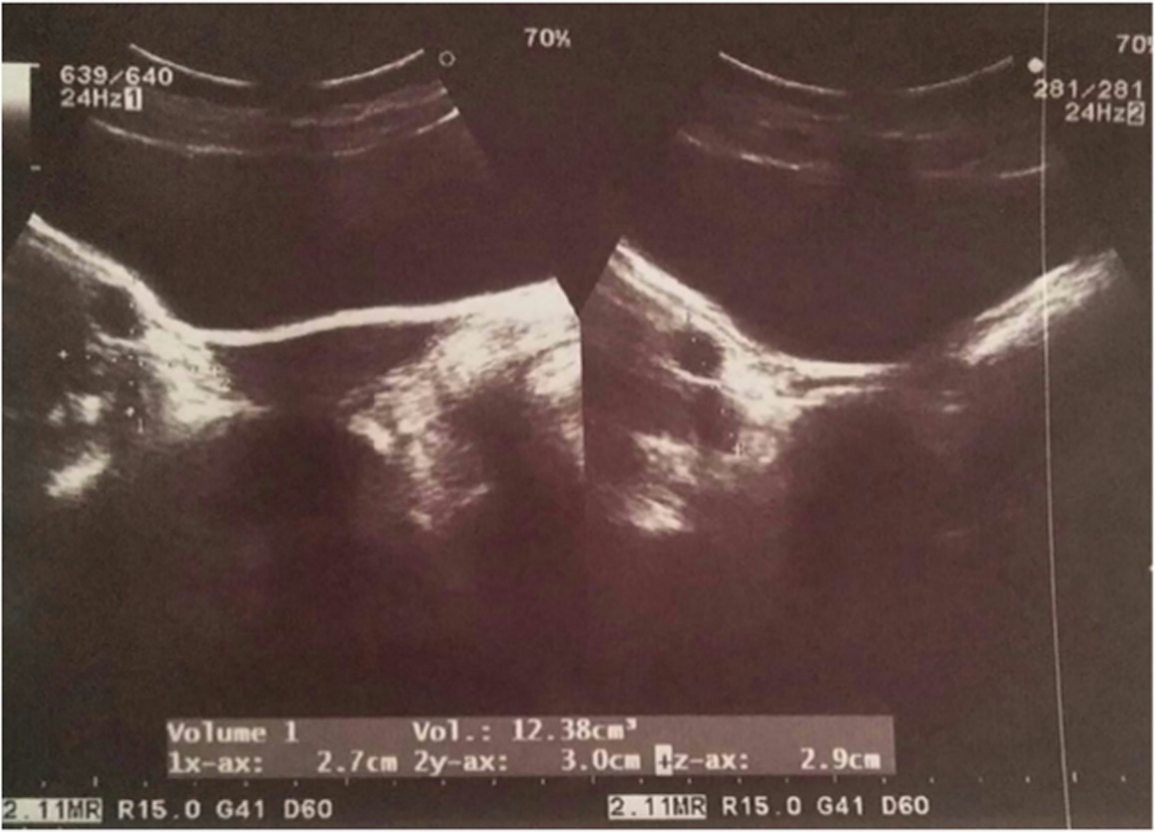
Trans‐abdominal ultrasound shows an enlarged right ovary with multiple cysts

Her transabdominal ultrasound showed a small uterus measuring approximately 6.7 cm × 1.8 cm × 1.7 cm with heteroechoic collection in the fundus and body of the uterus with thinning of the myometrium (Figure 1). Bilateral ovaries were found to be enlarged with multiple cysts (Figure 2). No inguinal masses were noted during the ultrasound.

**FIGURE 3:**
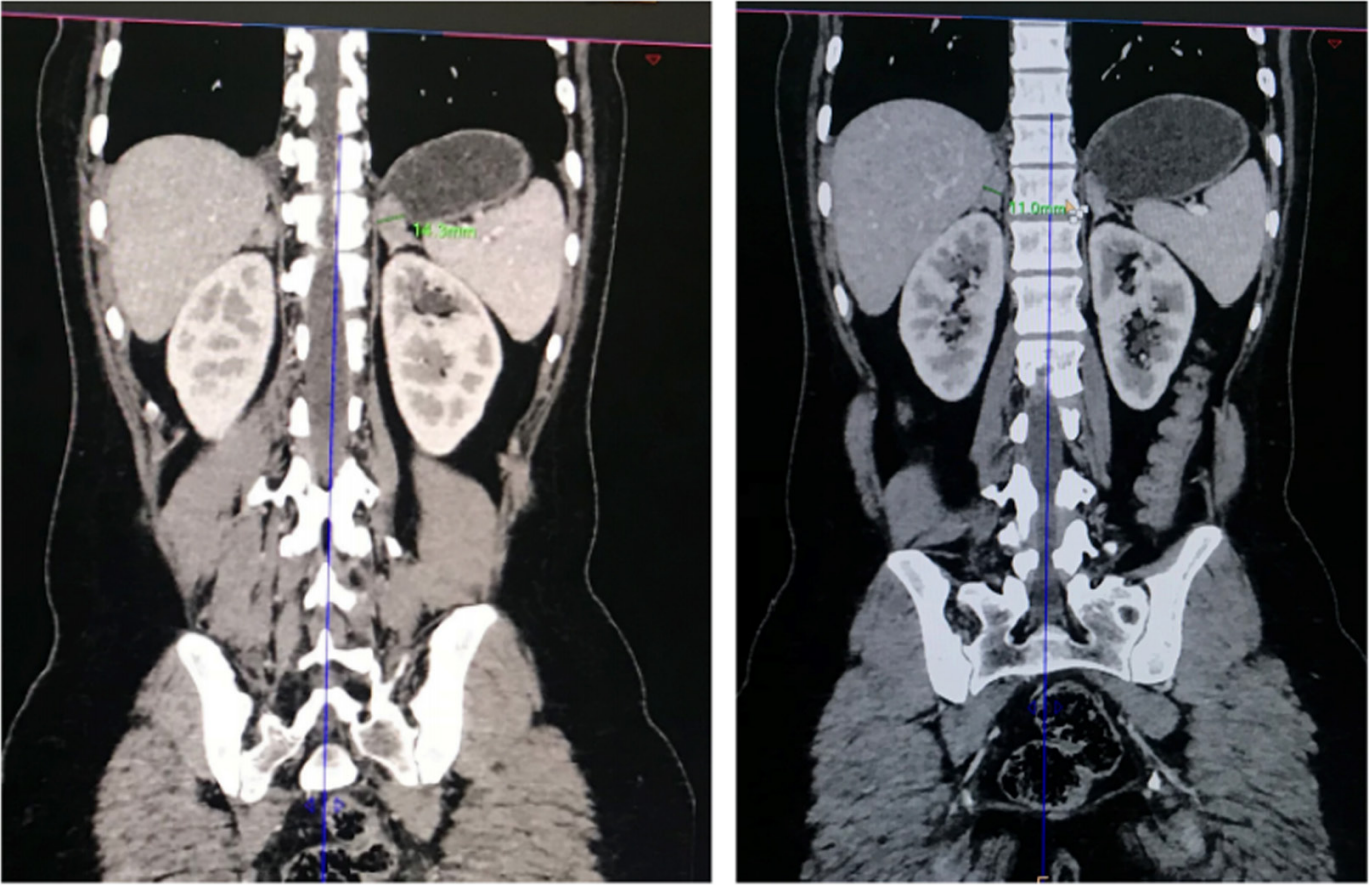
Coronal CT image showing bulky body of left and right adrenal glands measuring 14.3 mm and 11.0 mm, respectively

**FIGURE 4:**
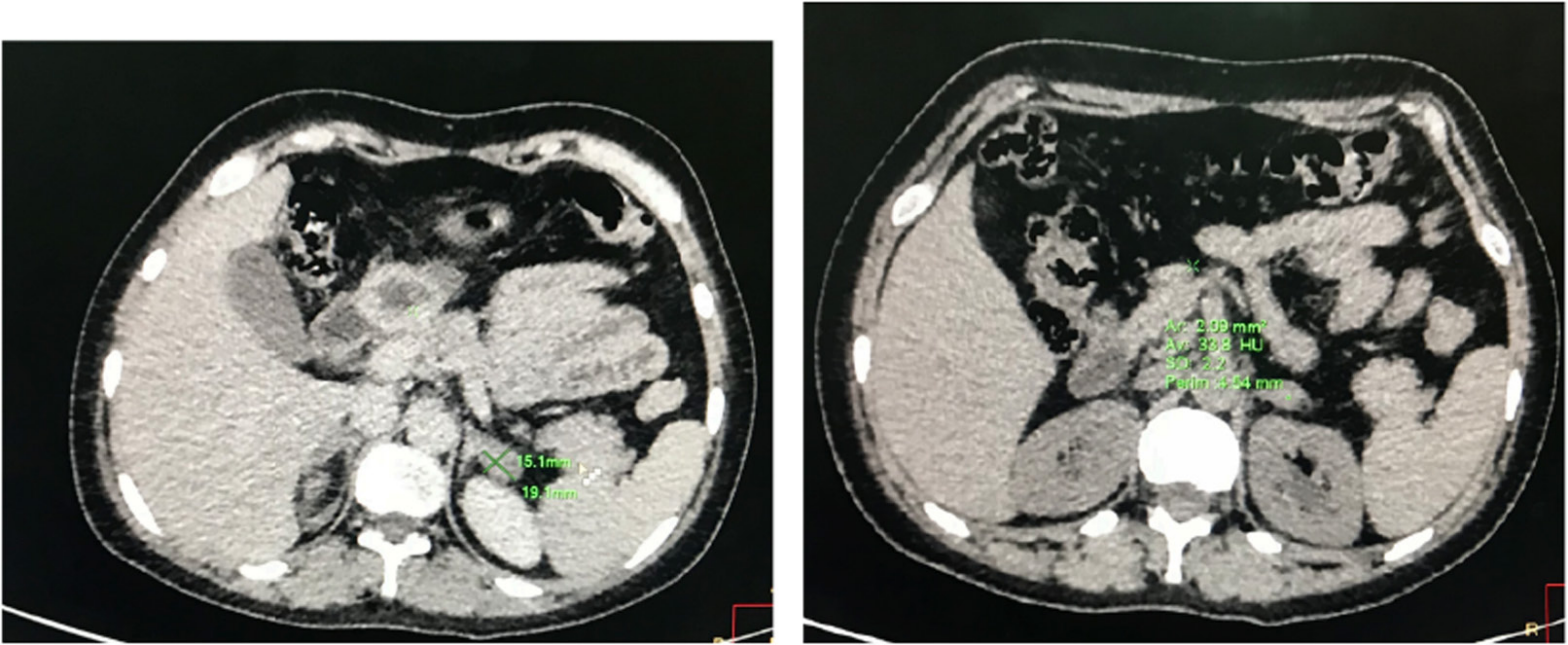
Axial non‐contrast CT image showing bulky left adrenal with a well‐defined nodular lesion in the lateral limb. No evidence of calcifications or fat attenuating areas within

**FIGURE 5:**
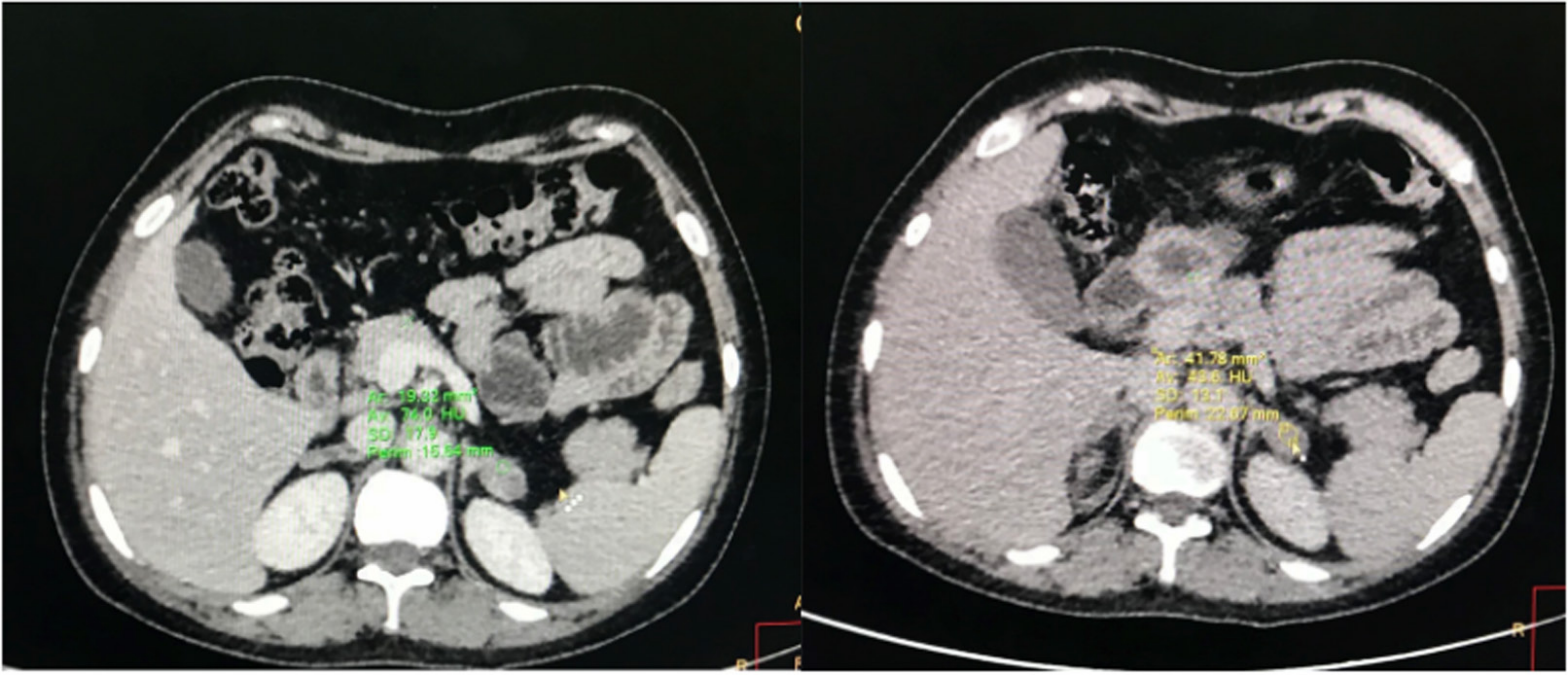
Axial CT image showing bulky left adrenal with a well‐defined homogenous nodular lesion in the lateral limb. On portal venous phase, the lesion shows homogenous enhancement (HU+74), and on delayed phase, it shows washout of contrast (HU+43) with respect to the portal venous phase

**FIGURE 6:**
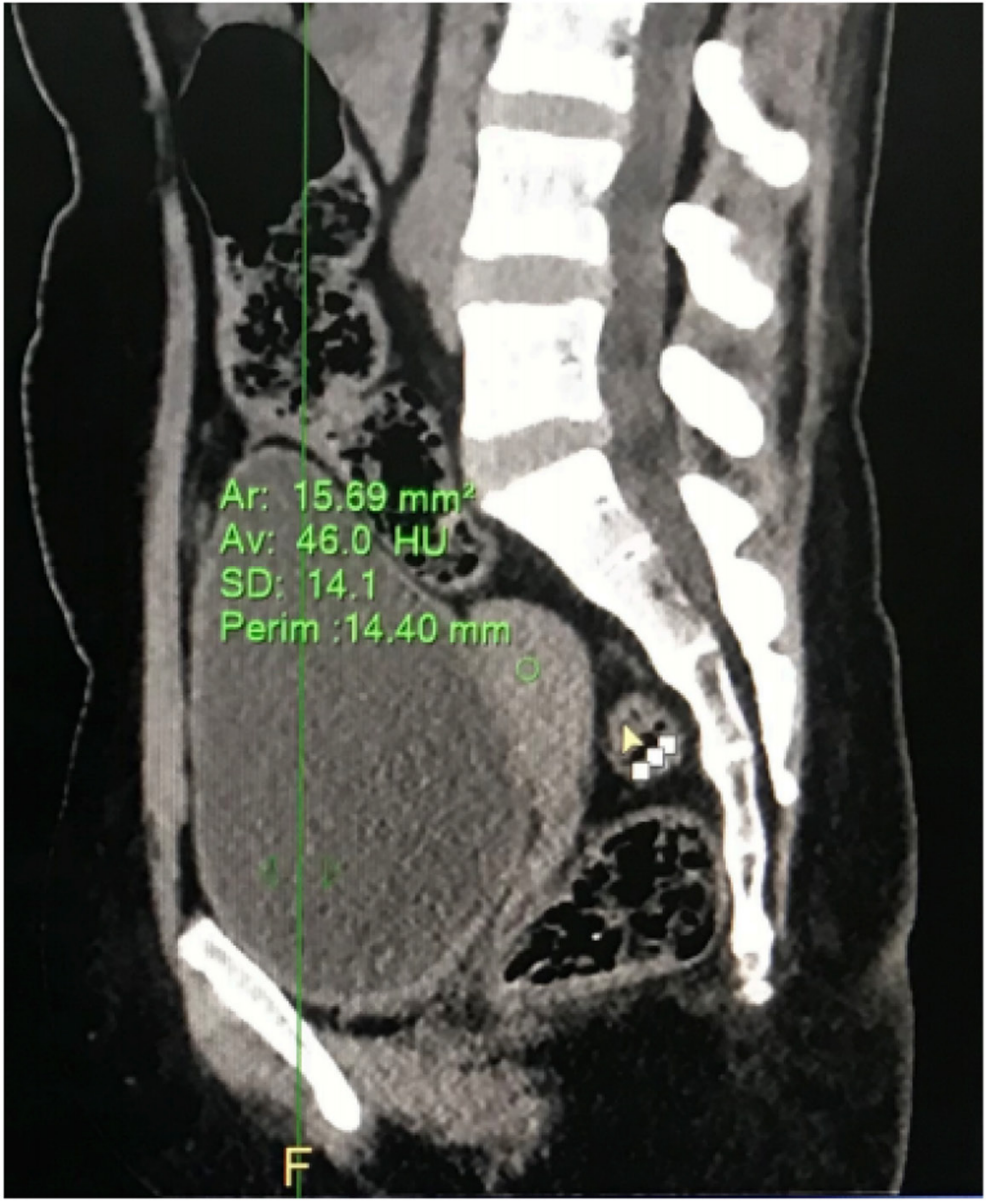
Sagittal CT image showing small sized uterus with diffusely thinned out endometrium and collection within endometrial cavity (HU+46)

**FIGURE 7:**
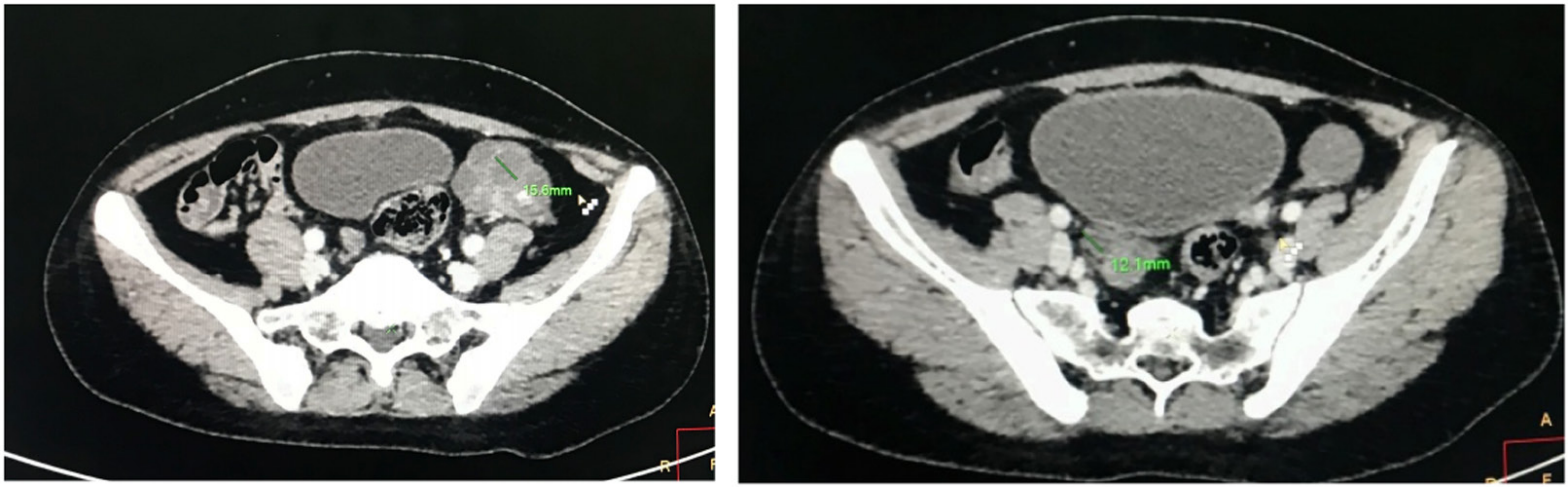
Axial CT image showing multiple non‐enhancing cystic lesions in bilateral ovaries, the largest measuring 15.6 mm and 12.1 mm in the left and right ovaries, respectively

A contrast‐enhanced computerized tomography (CECT) of the abdomen and pelvis was ordered. The CECT under adrenal protocol showed enlarged bilateral adrenal glands. The width of the body of the right and left adrenal glands measured 11 mm and 14.3 mm, respectively (Figure 3). The width of the lateral limb of the right adrenal gland measured 6.8 mm and that of the medial limb was normal at 5.9 mm. A well‐defined homogenous nodular enlargement measuring 15 × 19 mm was noted in the lateral limb of the left adrenal gland (Figure 4). On non‐contrast imaging, the nodule showed Hounsfield Units (HU) of +33 without any evidence of calcification or any fat density areas. On the portal phase of post‐contrast imaging, the nodule showed significant homogenous enhancement with an HU of +74, while on delayed imaging, the nodule showed an HU of +43 (Figure 5) (with absolute washout of 75% and relative washout of 41%). CECT also showed a small uterus with a hyperdense collection of HU +46 in the fundus and body and with thinning of the myometrium (Figure 6). Bilateral ovaries were enlarged with multiple cysts of variable sizes (Figure 7).

Based on her history, examination, and suggestive imaging features, the clinicoradiologic diagnosis of congenital adrenal hyperplasia was made. Due to limited resources and financial constraints, enzyme analysis and karyotype could not be done. The absence of salt‐wasting or virilization features and the presence of hypertension, cystic ovaries, and normal secondary sexual characteristics suggest the subtype of CAH to be of a partial deficiency of 17‐alpha‐hydroxylase enzyme, which is a rare occurrence.^8^, ^10^

## 3 DISCUSSIONS

Congenital adrenal hyperplasia (CAH) represents various enzyme deficiencies affecting the steroid hormone synthesis pathway in the adrenal cortex and ovaries in females. While the different enzymatic subtypes of CAH result in a universal decrease in the level of glucocorticoids and an increase in the level of glucocorticoid precursors, the level of mineralocorticoids and sex hormones is variable depending on the deficient enzyme.^12^ The decreased level of the glucocorticoid cortisol results in a loss of its negative feedback, thereby increasing the level of adrenocorticotrophic hormone (ACTH) secreted from the anterior pituitary. As ACTH has a trophic effect on the adrenal cortex, it results in hyperplasia of the cortex, thus explaining the etymology.^1^ The 21‐hydroxylase deficiency may present in a classical salt‐wasting or a nonclassical virilizing form,other types of CAH present with hypertension and other associated abnormalities.^12^ In this case, the absence of salt‐wasting features and the presence of hypertension rule out classical 21‐hydroxylase deficiency as the potential cause behind CAH. Similarly, the absence of features of virilization and the presence of symptoms of hypoestrogenism rule out 11‐beta‐hydroxylase and nonclassical 21‐hydroxylase deficiency as the causes.

In the common 21‐hydroxylase type of CAH, mineralocorticoids are low whereas androgens are high, thereby resulting in either classical salt‐wasting type or nonclassical virilizing clinical features.^12^ However, the rare forms of CAH include 11‐beta‐hydroxylase and 17‐alpha‐hydroxylase subtypes, which present with hypertension due to an increase in mineralocorticoid effect.^12^ The level of androgens is high in 11‐beta‐hydroxylase deficiency, whereas it is low in 17‐alpha‐hydroxylase deficiency. Although enzyme assay should be done biochemically, we had to rely on clinical and radiological diagnosis because of limited resources.

17‐alpha‐hydroxylase deficiency is a rare type of CAH. While the precursors are uninterruptedly propagated toward aldosterone synthesis, the formation of cortisol and androstenedione is decreased. Androstenedione is the precursor in the formation of testosterone. The first and second steps of the steroid biosynthetic process lead to an eventual formation of progesterone, whereas the formation of estrogen requires androstenedione to be converted to testosterone followed by eventual aromatization to estrogen.^12^ Hence, progesterone level is higher in 17‐alpha‐hydroxylase deficiency whereas that of estrogen is lower. As estrogen is the major female sex hormone responsible for negative feedback on the hypothalamus, lower estrogen levels result in the loss of negative feedback and an eventual rise in the levels of FSH and LH.^7^ The rise in FSH results in increased folliculogenesis and cystic ovaries as described in this case. Besides, as the level of LH is higher even at baseline and an initial increase in estrogen is required to provide a stimulus for the LH surge to occur just before ovulation, such a surge cannot occur in this case, resulting in anovulatory amenorrhea and subsequent infertility.^6^

The clinical and radiological findings seen in this case resemble that of another case reported in the literature by Kim et al.^11^ The case is that of an 18‐year hypertensive female who was otherwise asymptomatic. Her imaging showed a hypoplastic uterus with thin endometrium and bilateral polycystic ovaries, which are similar to the findings of our case. Her biochemical analysis also showed elevated FSH and LH levels and decreased cortisol levels. She later underwent karyotyping, ACTH stimulation test and genetic analysis which revealed a 46,XX karyotype, no change in basal serum 17 hydroxyprogesterone level after ACTH administration, and CYP17A1 gene mutation, respectively, thereby effectively diagnosing her as a case of 17‐alpha‐hydroxylase deficiency.

The normal size of the right and left adrenal bodies in their maximum dimensions is 8 mm and 10 mm, respectively.^2^ In this case, as the bulk of both adrenal glands exceeds their maximum dimensions, this supports hyperplasia as the probable cause. A solitary adenoma in an adrenal gland would have provided strong negative feedback due to the continuous production of hormones and thus would have caused atrophic changes in the remaining adrenal glands. In our case, the well‐defined homogenously nodular enlargement of adrenal glands without any atrophic changes in the remaining parts of both adrenal glands effectively rules out solitary adrenal adenoma as the cause of these findings. The gland shows no evidence of calcifications or fat attenuating areas within the nodule. With an HU value of below 10, this rules out lipid‐rich adenoma as the cause and makes macronodular adrenal hyperplasia a more likely cause.^3^

The HU values of the adrenal lesion are +33, +74, and +43 in non‐contrast, post‐contrast, and delayed contrast phases, respectively. This gives an absolute washout value of 75% and a relative washout value of 41%. This makes adrenal carcinoma a lesser likely cause and rules the diagnosis in favor of adrenal adenoma or adrenal hyperplasia.^4^

A limb width of more than or equal to 5 mm is 100% specific for diagnosing bilateral adrenal hyperplasia. As the limb width of bilateral adrenal glands in this case is more than 5 mm, this condition can be diagnosed as bilateral adrenal hyperplasia with 100% specificity. (Allison).

Besides, as the growth of the uterus relies on the monthly cycling of estrogen and progesterone, the defect in estrogen formation due to CAH results in a failure of the endometrium to grow during the proliferative phase. In the lack of estrous uterus, the mere presence of progesterone is not helpful to turn it into secretory endometrium. This results in an atrophic uterus as in this patient.

## 4 CONCLUSION

Congenital adrenal hyperplasia is a rare biochemical disorder with various clinical manifestations. The manifestations vary according to the enzyme affected and the place in the pathway where the deficient enzyme works. The distortion in the biochemistry can affect other organ systems as well, especially the ovaries and the uterus in females. Radiological evidence can be employed to diagnose this condition.

## AUTHOR CONTRIBUTION

HK observed and supervised the entire authorship process. SB reviewed the literature and contributed to drafting and editing the manuscript. PD and SS collected all the required information, images, and reports, reviewed the literature, and contributed to drafting the manuscript.

## CONFLICTS OF INTERESTS

The authors declare that they have no competing interests.

## ETHICAL APPROVAL

This study did not include experiments on animals or humans.

## CONSENT

Written informed consent was obtained from the patient to publish the report in accordance with the journal's patient consent policy.

## Data Availability

The data that support the findings of this study are available on request from the corresponding author.
